# lmmuno Histochemical Profile of Endometrium in Patients With Genital Endometriosis

**DOI:** 10.1155/DTE.3.127

**Published:** 1997

**Authors:** L. Mettler, A. Jürgensen, N. I. Volkov, V. Kulakov, M. R. Parwaresch

**Affiliations:** 1 Department of Obstetrics and Gynecology University of Kiel Michaelisstrasse 16 Kiel 24105 Germany; 2 Institute of Cytopathology University of Kiel Michaelisstrasse 16 Kiel 24105 Germany; 3 Research Centre of Obstetrics, Gynecology and Perinatology Oparin Street Moscow 117815 Russia; 4 Institute of Pathology University of Kiel Michaelisstrasse 11 Kiel 24105 Germany

## Abstract

The aim of present study was to investigate the occurence of different lymphocyte subsets in the endometrium of endometriosis patients and in healthy women on every day of the menstrual cycle with special emphasis to the proliferative activity of endometrial cells with Ki-S3 antibody. We also conducted immunohistochemical studies of T-lymphocytes, B-lympho-cytes, macrophages, natural-killer-cells and also of antigens class II of the histocompatibility complex (HLA-DR) during the different phases of the menstrual cycle in endometriosis and non-endometriosis patients.

Endometrial lymphocyte subsets show equal quantity and distribution in endometriosis patients and in the control group. After a peak in the early preoliferative phase the absolute number of T-lymphocytes decreases while a predominance of T-suppressor/cytotoxic T-lymphocytes (CD8) compared to T-helper/inducer lymphocytes (CD4) occurs towards the end of the menstrual cycle.

It can be concluded that endometrium as the potential parent epithelia of endometriotic lesions seems not to be altered in its lymphatic cell content compared to healthy women. Furthermore endometrium is clearly characterised as part of the mucosa associated lymphatic tissue (MALT). T-lymphocytes show specific quantitative changes due to different phases of the menstrual cycle.


*Diagnostic and Therapeutic Endoscopy*, 1997, Vol. 3, pp. 127–145
Reprints available directly from the publisher
Photocopying permitted by license only

© 1997 OPA (Overseas Publishers Association)
Amsterdam B.V. Published in The Netherlands
by Harwood Academic Publishers
Printed in Singapore

### Immuno Histochemical Profile of Endometrium in Patients with Genital Endometriosis

L. METTLER,[1] A. JÜRGENSEN,[2] N. I. VOLKOV,[3] V. KULAKOV[3] and M. R. PARWARESCH[4]

[1]*Department of Obstetrics and Gynecology, University of Kiel Michaelisstrasse 16, 24105 Kiel/Germany,* [2]*Institute of Cytopathology, University of Kiel Michaelisstrasse 16, 24105 Kiel/Germany,* [3]*Research Centre of Obstetrics, Gynecology and Perinatology* [4]*Oparin Street, 117815 Moscow/Russia,* [4]*Institute of Pathology, University of Kiel Michaelisstrasse 11, 24105 Kiel/Germany*

*(Received 29 January 1996; revised 17 June 1996; in final form 3 July 1996)*

**The aim of present study was to investigate the occurence of different lymphocyte subsets in the endometrium of endometriosis patients and in healthy women on every day of the menstrual cycle with special emphasis to the proliferative activity of endometrial cells with Ki-S3 antibody. We also conducted immunohistochemical studies of T-lymphocytes, B-lympho-cytes, macrophages, natural-killer-cells and also of antigens class II of the histocompatibility complex (HLA-DR) during the different phases of the menstrual cycle in endometriosis and non-endometriosis patients.**

**Endometrial lymphocyte subsets show equal quantity and distribution in endometriosis patients and in the control group. After a peak in the early preoliferative phase the absolute number of T-lymphocytes decreases while a predominance of T-suppressor/cytotoxic T-lymphocytes (CD8) compared to T-helper/inducer lymphocytes (CD4) occurs towards the end of the menstrual cycle.**

**It can be concluded that endometrium as the potential parent epithelia of endometriotic lesions seems not to be altered in its lymphatic cell content compared to healthy women. Furthermore endometrium is clearly characterised as part of the mucosa associated lymphatic tissue (MALT). T-lymphocytes show specific quantitative changes due to different phases of the menstrual cycle.**

*Keywords:* Immunohistochemical profile of endometrium, genital endometriosis, lymphocyte subsets

### INTRODUCTION

Endometriosis is a disease that primarily affects women within the reproductive age. Many efforts have been made to explain its pathogenesis as metastatic, metaplastic or on the basis of some immunological mechanisms. Retrograde menstruation is accepted as a source of endometrial

To whom the correspondence should be addressed: Mettler, L., Department of Obstetrics and Gynecology, University of Kiel Michaelisstrasse 16, 24015 Kiel/Germany. Fax: 49-431-597 2149.

cells found in the peritoneal cavity [8]. This is recognized as a common phenomenon but it is still not known why only a small cohort of women with patent Falloppian tubes suffer from endometriosis. Numerous approaches have been made in an effort to answer this question. During the last years many investigators demonstrated the association of endometriosis and changes in the immune system which were elucidated as alterations in cell-mediated as well as humoral immunity [5,20].

The results suggest that the immune system might play a substantial role in the pathogenesis of endometriosis and associated infertility. At the same time the role of the immune system in the pathogenesis of endometriosis and infertility remains unclear.

We have undertaken this study in order to elucidate whether or not there is any difference between the proliferative activity of endometrial cells in women with endometriosis and without it and to see weather or not there is a difference in occurence and distribution of immunocompetent cells in the endometrium. To answer this question we investigated the proliferative activity of endometrial cells with Ki-S3 antibody. We conducted immunohistochemical studies of T-lymphocytes, B-lymphocytes, macrophages, natural-killer-cells and also of antigens class II of the histocompatibility complex (HLA-DR) during the different phases of the menstrual cycle.

### MATERIAL AND METHODS

Endometrial specimen were obtained by biopsy from 253 patients with regular (28–29 days) ovulatory menstrual cycles (proved by basal body temperature curves and blood progesterone levels). All women underwent laparoscopy and endometrial tissue biopsy with the framework of infertility work up. No one had received any medical treatment during the previous three months.

The main group consisted of 143 patients with endometriosis stages I–III (Fig. 1–3) (according to the revised AFS classification [Americal Fertility Society 1985]) proved by direct laparoscopic classification and partially by biopsy of lesions. The patency of tubes were proved laparoscopically in all the patients. The mean age of the patients of the group was 29.8 years (from 24 to 36), and the mean infertility period was 6.8 years (from 3 to 14 years).

The control group included 110 patients with laparoscopically proven tubal factor infertility resulting from the prior pelvic inflammatory disease (PID). All patients had previously undergone antibiotic therapy. Although the tubes were blocked partially in all the cases, there were no signs of inflammatory process in progress at the moment of laparoscopic investigation. Fimbrioplasties and salpingostomies (Fig. 4–6) were performed frequently. The mean age of the patients in the control group was 29.6 years (from 24 to 35 years), the mean infertility period — 6.3 years (from 2 to 14 years).

Operative laparoscopy = pelviscopy and chromopertubation [14] was performed in all patients of both groups. The distribution over the menstrual cycle revealed an incidence up to 7 patients per day. Within the framework of this infertility protocol endometrial biopsies were

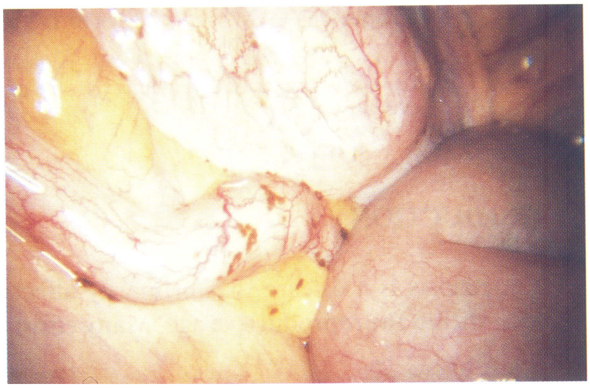

FIGURE 1 Pelviscopic site of endometriotic lesion on the appendix (AFS I)

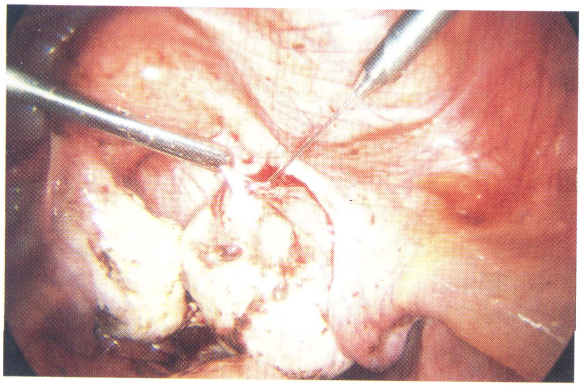

FIGURE 2 Pelviscopic site of endometriotic lesions on the surface of the ovary (AFS II).

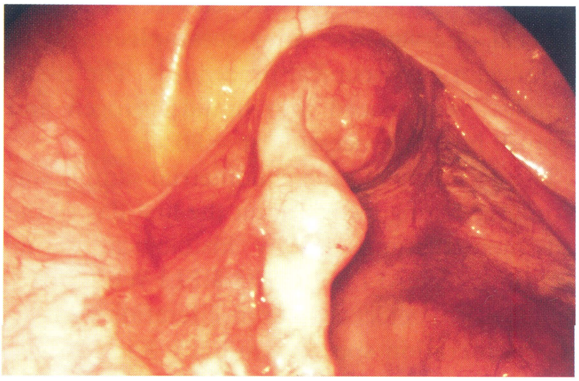

FIGURE 4 Pelviscopic site of endosalpinpitis nodosa.

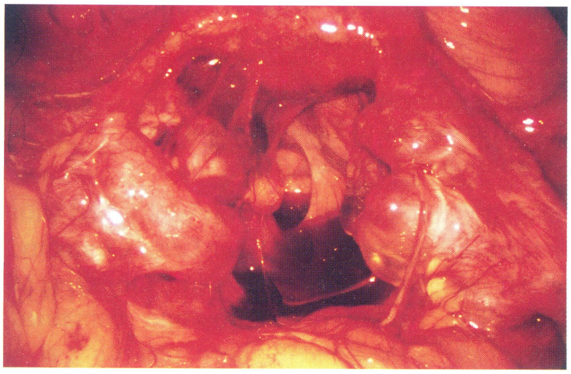

FIGURE 3 Ovarian endometriotic cyst (AFS III) at pelviscopy.

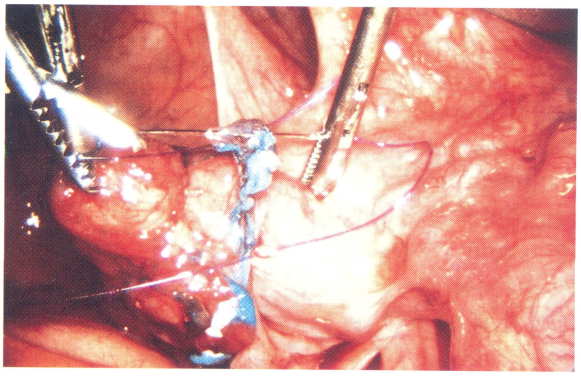

FIGURE 6 Suturing of fimbria with intracorporal knotting technique — pelviscopic site.

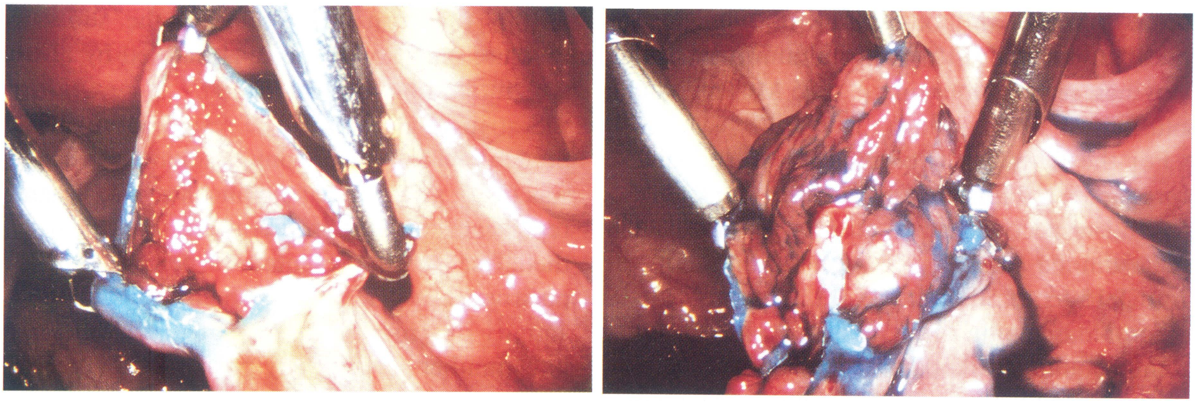

FIGURE 5 Terminal salpingostomy and eversion of fimbria by pelviscopy.

taken from all patients at the end of the laparoscopical interven tion after accomplishment of all relevant endoscopical procedures. The tissue samples were placed into liquid nitrogen and kept till further processing at - 80°C.

The cycle day of each endometrial sample was estimated taking into account the first day of the last menstruation and by histological dating according to DALLENBACH-HELLWEG [4].

In order to detect cellular proliferation we used the proliferation specific antibody Ki-S3 which recognizes a new epitope on the KI-67 nuclear antigen [17]. The Ki-S3 antibody shows no coreactivity with non proliferation related structures especially no coreaction with cytoplasm. The appropriate number of cryostate sections 5 µm thick (Equipment: Frigo-Cut Mod 2700, Fa. Reichert-Jung, Nussloch) were prepared for immunochemical investigation with Ki-S3 antibodies. Serial cryostat sections were mounted on clean glass slides and air dried for 2 hours at room temperature. Then sections were fixed for 5 minutes in acetone, dried and stored at - 80°C till further processing.

The indirect immunoperoxidase technique modified by STEIN *et al.* [6] allowed to obtain brown-coloured reaction products in the area of antigen bindings. The primary antibodies used with their antigen determinant, cluster designation, related dilution, and sources are shown in Table I.

For staining the slides were incubated with the diluted primary antibodies at room temperature for 30 minutes in 1% Ringeralbumin solution (Bovines serum albumin in 0.05 M Tris/NaGl solution pH 7.4).

For the following two colour steps the slides were incubated for half an hour with the secondary and respectively third antibodies which had to be diluted in 10% human albumin solution from 1:15 to 1:150 (pH 7.4).

Peroxidase-conjugated rabbit-anti-mouse immunoglobulin (Dakopatts/Denmark) was used as the secondary antibodies, and peroxidase-conjugated goat-anti-rabbit immunoglobulin (Danova, Hamburg) was used as the third antibodies.

All slides were thoroughly washed several times between these two particular colour steps with 0.05 Tris/NaCl solution (pH 7.4). The peroxidase-developing reaction was observed from 5 to 10 minutes with 3′, 3′ diaminobenzidin-tetrachloride (0.6 mg/ml; Fa. Walter, Kiel) and $H_2O_2$ (0.01 Vol %) in Tris/HCl-bufferat pff 7.6. For the light microscopic investigation the slides were counterstained with haemalaun and stocked up with glycerin-gelatine (Fa. Merck, Darmstadt).

The cryostat slides of human tonsils were used to a certain optimal antibody dilution and for positive control. For negative control the primary antibody was replaced by PBS-buffer.

### Light Microscopy of Endometrium for Quantitation of Lymphocytes

The analysis and evaluation was performed with the light microscope (SM Lux, Leitz Company, Wetzlar using an Ocular of × 25 and Objective of × 40). The ocular of microscope contained square rasters designed to keep regarding at a magnification during the determination of the absolute number of marked cells.

The fields were evaluated for each slide Corresponding to the magnification × 250 one real field was 1.85 $mm^2$, and to the magnification × 400 - 0.76 $mm^2$. For each of the slides the absolute quantity of the cells marked by respective antibodies were calculated.

Statistical analysis of the results was made by Student's T-test. The conventional level of 0.05 was taken as a limit of significance. For diagram representation the proliferation activity of epithelial cells was presented as percent of Ki-S3 positive cells per 1000 epithelial cells while the activity of stromal cells was considered as absolute number of antibody-positive cells per one square millimeter.

TABLE I Applied monoclonal antibodies for immunohistochemistry

| Name of monoclonal antibody | Cluster-designation (CD)* | Specificity of epitop | Occurrence of antigen | Dilution | Source of antibody |
|---|---|---|---|---|---|
| Anti-LEU-4 | CD 3 | molecular weight (MW) 22–28 kD, surface antigen | T-lymphocytes, pan-T-marker | 1:200 | Becton-Dickinson, USA; No. 7340 |
| Anti-Leu-3a | CD 4 | MW 55 kD, surface-antigen | $T_4$-helper/inducer-lymphocytes; [monocytes; macrophages] | 1:400 | Becton-Dickinson, USA; No. 6320 |
| Anti-Leu-2a | CD 8 | 1. MW 32 kD 2. MW 43 kD surface-antigen | $T_8$-cytotoxic/ suppressor-lymphocytes | 1:400 | Becton.-Dickinson, USA; No. 6310 |
| Anti-Leu-7, (HNK-1) | | MW 110 kD | Natural Killer cells (NK); | 1:50 | Becton Dickinson, USA, No. 7390 |
| Anti-Human-B-Cell, Clone To 15 | CD 22 | MW 130 kD | B-lymphocytes, pan-B-marker | 1:100 | Dakopatts, Denmark No. M708 |
| Anti-Human-HLA-D, Clone CR3/43 | | MW 28 kD beta-chain of gene products of HLA-DP, DQ and -DR** | B-lymphocytes, macrophages, activated T-lymphocytes dendritic cells | 1:500 | Dakopatts, Denmark, No. M775 |
| Ki-M 1P | | MW 60 kD | histiocytes, blood monocytes | 1:5.000–10.000 | Inst. of Pathol., Kiel, Germany |
| Ki-M 6 | CD 68 | MW 60 kD; intracytoplasmat antigen; surface membrane of phagosomes and lysosomes | histiocytes, blood monocytes | 1:5.000 | Institute of Pathology Kiel, Germany |
| Ki-M 8 | | 1. MW 30 kD 2. MW 32 kD; intracytoplasmat antigen surface membrane of phagosomes and lysosomes | histiocytes, blood monocytes | 1:5000 | Institute of Pathology Kiel, Germany |
| Ki-S 3, | | 1. MW 340 kD 2.MW390 kD; nuclear Antigen | proliferating cells in cell cycle phases G1, S, G2, M | 1:8.000 | Institute of Pathology Kiel, Germany |

*III. International Workshop on Human Leukocyte Differentiation Antigens, Oxford 1986.
**International Workshop on Monoclonal Antibodies to Human MHC Class II Antigen, 1983.

### RESULTS

### General Data

There were no endometrial structural abnormalities.

### *Proliferative Activity of Superficial Epithelial Cells*

At the early proliferation phase of the control group the Ki-S3 positive of superficial epithelial cells was as high as 36%. Then it slightly increased to 38% on day 10 of the menstrual cycle and afterwards it slowly decreased to Zero on day 26 of the cycle. After day 26th no Ki-S3 positive cells were found in superficial epithelia. In the endometriosis group 40% of superficial epithelial cells of endometrium were found as Ki-S3-positive in the early follicular phase and the other phases followed the pattern of the control group.

The dynamics of Ki-S3 positivity during the entire menstrual cycle are seen in Fig. 7A.

The slight differences were found as statistically not significant and variable at all the points ($p > 0.05$).

### *Proliferative Activity of Glandular Epithelium*

In the control group the percentage of Ki-S3 positive cells ascended dramatically from 7% on day 5 of the cycle to 57% on day 10 of menstrual cycle. The level of glandular cells positivity was as high (with a slight decreasing to 30% on day 12 of the cycle) till the 15th day and afterwards the proliferative activity decreased quite rapidly to the 24th day of menstrual cycle.

After the 25th day of the cycle no more proliferative activity of glandular cells was found according to Ki-S3 positivity.

The Ki-S3 positivity in the endometriosis group demonstrated the same pattern.

Comparing the percentage of proliferative activity during menstrual cycle in the glandular epithelium in both groups no statistical difference ($P > 0.05$) could be detected. The course of Ki-S3 positivity in both control and endometriosis groups was very similar (Fig. 7B).

### *Proliferative Activity of Stromal Cells*

In the control group the number of Ki-S3 antibody marked cells demonstrated a rapid increase from 200 cells per $mm^2$ on day 5 of the menstrual cycle up to 500 cells per $mm^2$ on day 10 of the cycle. After a slight decrease between day 11 and 13 a second elevation of proliferative activity of stromal cells reaches in average 700 Ki-S3 positive cells per 1 $mm^2$ of the stroma on day 16 of the cycle and a mean number of 430 activated cells per $mm^2$ on day 17 of the cycle. From that day the quantity of Ki-S3 positive cells fluctuates around 400 active cells per 1 $mm^2$ up to the end of menstrual cycle.

The same course with a negligible difference ($P > 0.05$) was found in respect of Ki-S3 positivity of stromal cells in the endometriois group at all points of the entire menstrual cycle (Fig. 7C).

The above described features of proliferative activity of superficial epithelial cells, glandular epithelial cells and stromal cells in the control group and in the endoometriosis group are presented in Fig. 8 demonstrating immunohistochemically treated tissue samples.

### T-Lymphocytes

### *Anti Leu 4 (CD3) Pan T-Lymphoctes (Figure 9A)*

In the control group the number of CD3 positive lymphocytes at the early proliferation phase started from values of 420 cells per $mm^2$ on day 5 of the menstrual cycle to 570 CD3 positive cells per $mm^2$ on day 6 of the cycle ($P > 0.05$). After the 6th day of the cycle the number of CD3 positive cells decreased continuously to a mean value of 180 cells per $mm^2$ on day 12. Further on the CD3 positive cells remained relatively constant varying between 100–150

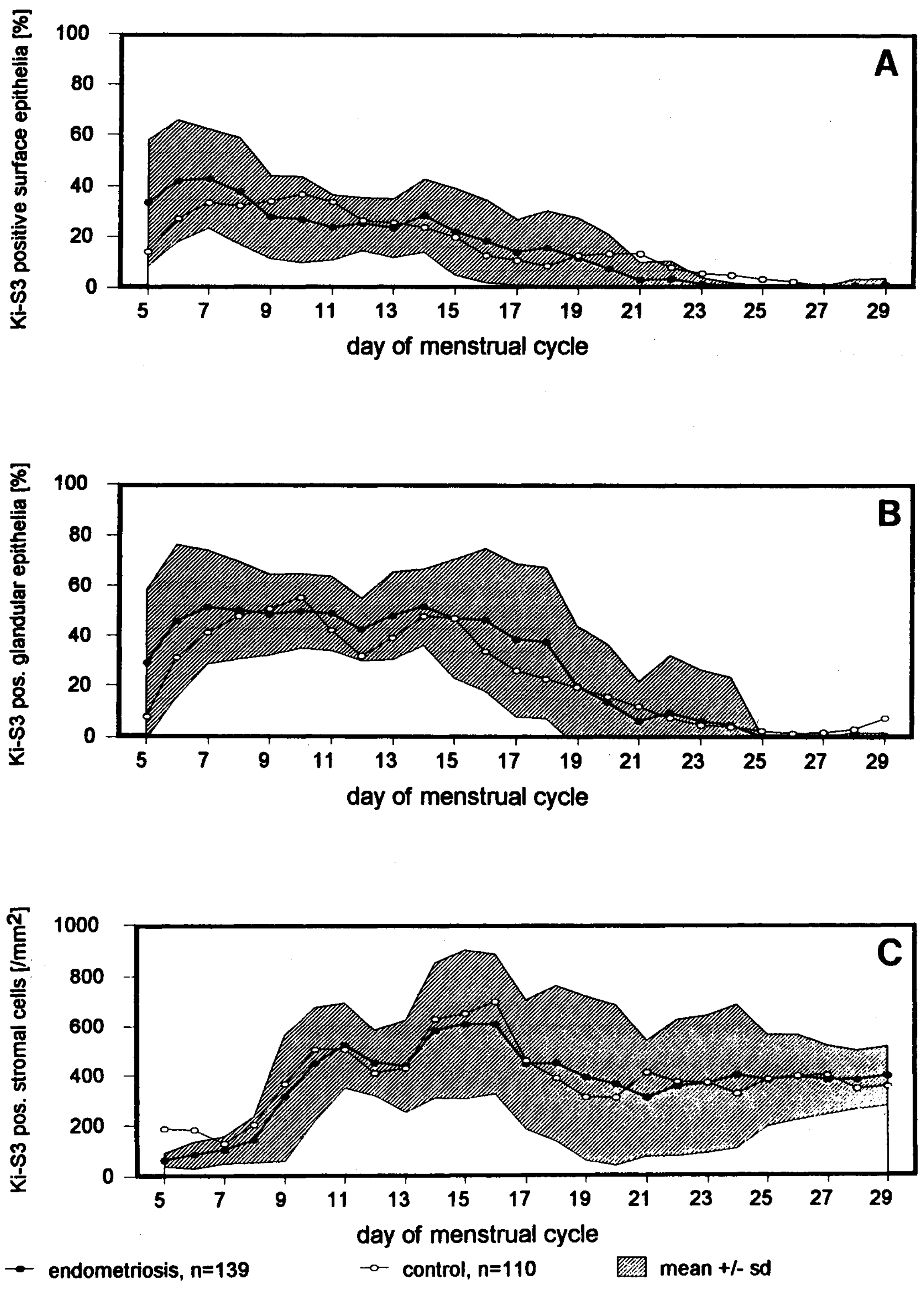

FIGURE 7 Proliferating endometrial surface epithelia (A), glandular epithelia (B) and stromal cells (C) in women with endometriosis compared to a control group throughout the menstrual cycle. [sd: standard deviation]

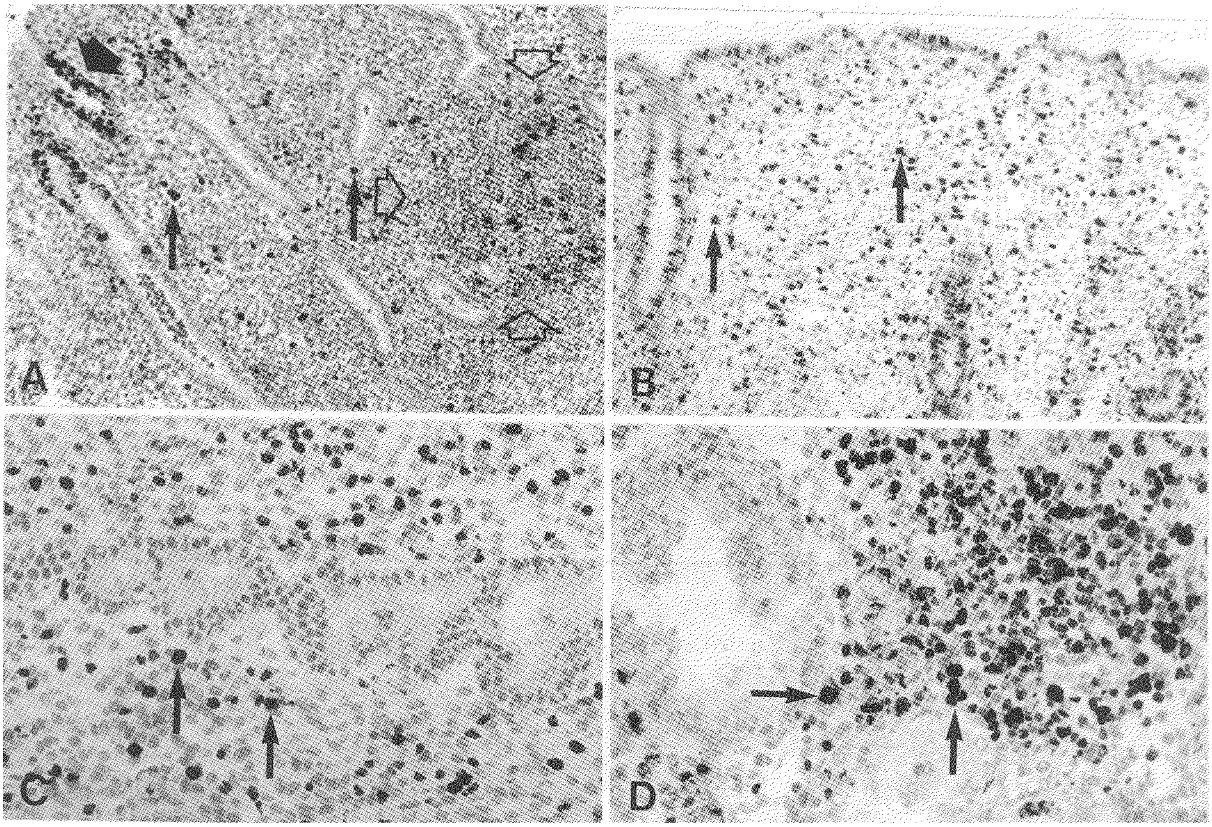

FIGURE 8 Ki-S3 as a marker of endometrial proliferation. In early follicular phase (A, × 350) few glandular epithelia (broad arrow) and single stromal cells (small arrows) showing proliferative activity. Note lymphocyte aggregation (open arrows) containing some proliferating cells. Maximum occurrence of Ki-S3 positive stromal cells (arrows) at time of ovulation (B, × 350). Complete arrest of proliferation in glandular epithelial in contrast to increasing numbers of Ki-S3 positive cells (arrows) in endometrial stroma on cycle day 23 (C, × 700). Lymphocyte aggregation in late secretory phase (D, × 700) demonstrating numerous proliferating cells.

cells per $mm^2$ till the end of the cycle. The comparison of the mean level and dynamic pattern of CD3 positive lymphocytes in endometrium during menstrual cycle demonstrated no significant differences in women with endometriosis compared to the control group almost on all days of the cycle ($P > 0.05$). Figure 10 demonstrates T-lymphocytes scattered in the endometrium around ovulation.

### *Anti-Leu 3a (CD4): Helper/Inducer T-Lymphocytes — Figure 9B*

In the control group the number of CD4 positive lymphocytes had the maximal value on days 5 and 6 of the menstrual cycle corresponding to 110–120 CD4 positive cells per $mm^2$ and rapidly decreased till the end of follicular phase to 30 T-lymphocytes per $mm^2$ on day 13 of the cycle. After an insignificant increase on day 16 it slightly decreased to the mean level of 20 CD4 positive cells per $mm^2$ ($P > 0.05$) up until the end of menstrual cycle.

In patients with endometriosis during the entire menstrual cycle the dynamic pattern of CD4 positive lymphocytes corresponded exactly to the course of the control group without any significant differences ($P > 0.05$).

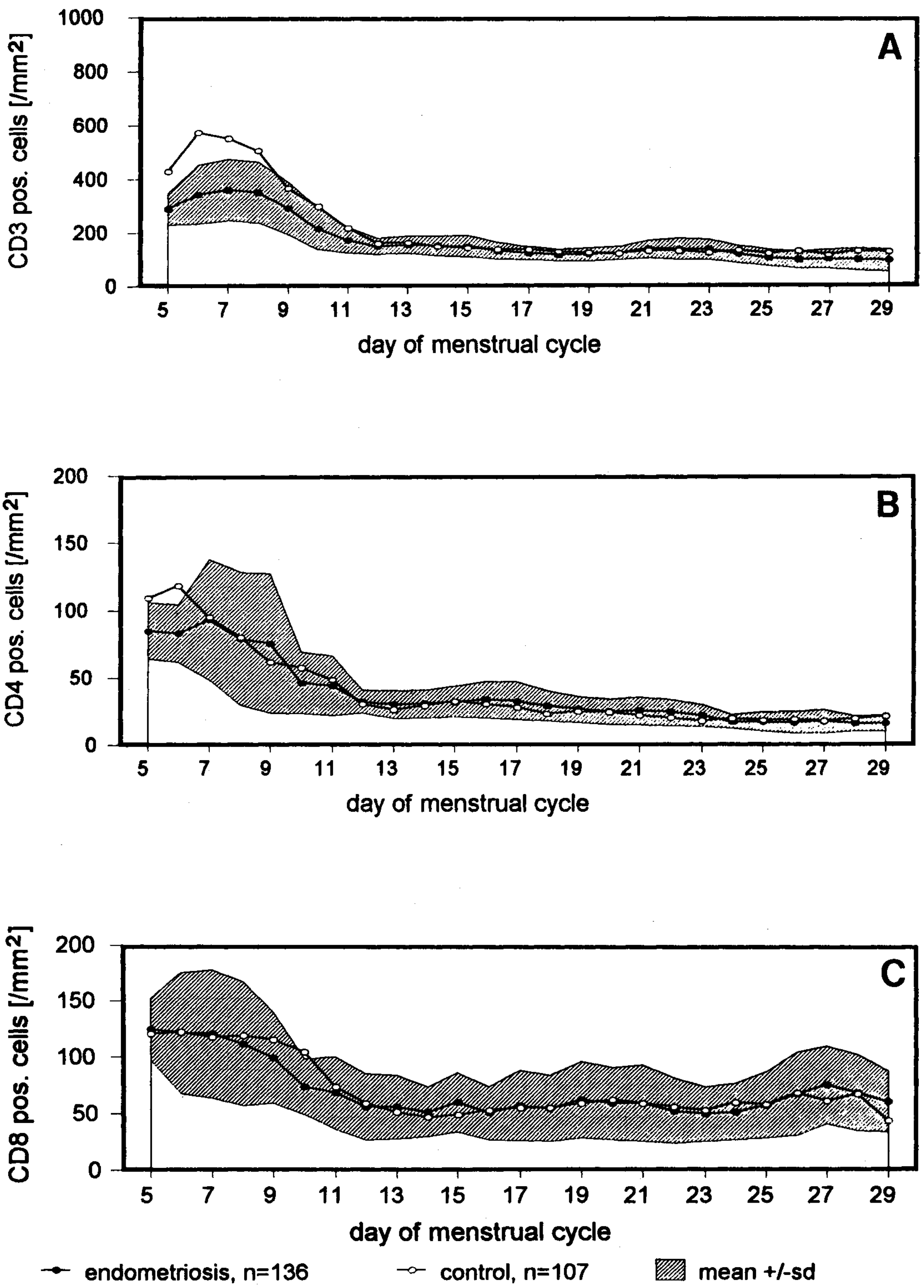

FIGURE 9 Different subsets of T-lymphocytes in the endometrium of women with endometriosis compared to a control group. [sd: standard deviation]

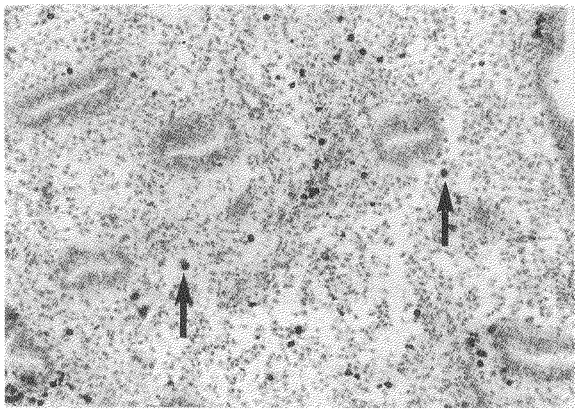

FIGURE 10 Scattered endometrial CD3 positive T-lymphocytes (arrows) in follicular phase ( × 350).

### *Anti-Leu 2a (CD8): Suppressor/Cytotoxic T-Lymphocytes — Figure 9C*

The maximal level of CD8 positive lymphocytes in the control group was found on days 5–8 of the menstrual cycle with 125 CD8 positive cells per mm$^2$ From day 9 of the cycle the number dramatically decreased to mean values of 48 cells per mm$^2$ on day 14 of the menstrual cycle. During the luteal phase of the cycle after day 14 the mean number of CD8 positive cells was relatively constant slightly but not significantly increasing towards the end of the cycle ($P > 0.05$).

In patients with endometriosis variations of CD8 positive lymphocytes quantity per mm$^2$ corresponded to the levels of those in the control group during the entire menstrual cycle. The differences were not statistically significant ($P > 0.05$).

### *Natural Killer Cells: Anti-Leu 7*

Throughout the entire menstrual cycle Leu 7 positive cells were noted as very scanty, weak coloured without special localization. These cells were found in endometrial stroma and in lymphocytes aggregations. Some slides demonstrated no Leu 7 positive cells. The maximal number of Leu 7 positive lymphocytes in the controls was registered as 20 cells per mm$^2$ on day 5 of menstrual cycle. In endometriosis patients the number of Leu 7 positive lymphocytes was constant during the entire menstrual cycle with negligable variations of the mean number from 0.6 to 4 Leu 7 positive cells per mm$^2$. The course of cells did not statistically differ from the controls ($P > 0.05$).

### B-Lymphocytes

### *Anti Human B-Lymphocytes — Figure 11*

They were almost exclusively detected in lymphocytes aggregations (Fig. 12). Similar microscopic pictures were found both in the control group and in patients with endometriosis.

The endometrium of endometriosis patients contained the maximal quantity of CD-22 positive cells on days 5–8 of the menstrual cycle (40 cells per mm$^2$). Then the number of CD-22 positive cells decreased to the end of proliferation phase to 20 cells per mm$^2$ on day 11 of the cycle and afterwards the mean values varied from 10 to 25 CD-22 positive cells with peak values of 20–21 cells on day 13.

No differences occured in the 2 investigated groups.

### Anti HLA-DR

### *HLA-DR Expression in the Endometrial Stroma*

HLA-DR positive cells were found in different layers of endometrium in patients of both groups: there was no real difference between the endometriosis group and the controls. In both groups the endometrial samples of basal stroma and lower basal layer contained HLA-DR positive cells with much density as compared to superficial endometrium (Fig. 13A).

HLA-DR was particularly pronounced at the late secretory phase. Endometrial stroma and lymphocytes aggregations consisted almost exclu sively of positive cells. The single HLA-DR pos-

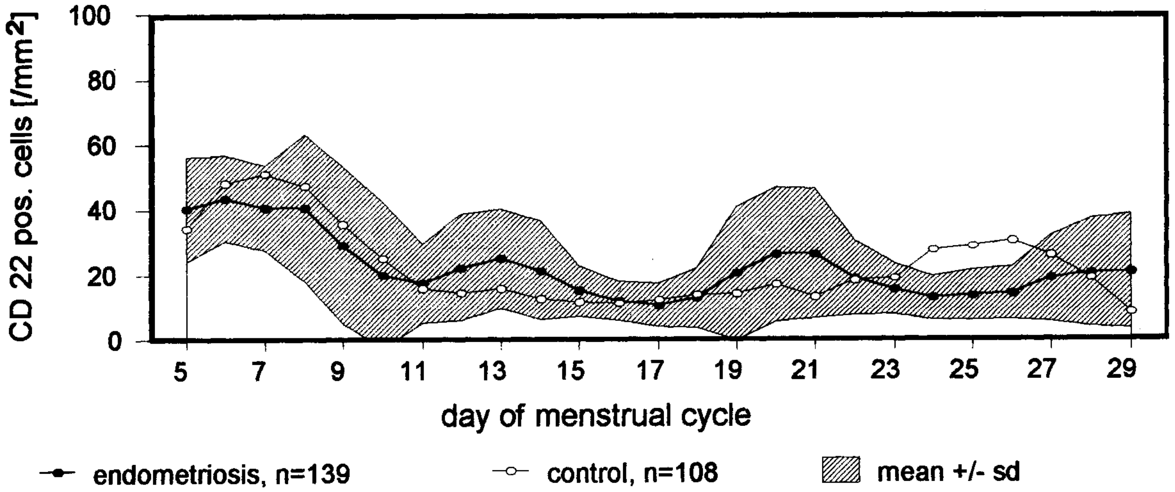

FIGURE 11 CD22 positive B-lymphocytes in the endometrium of women with and without endometriosis. [sd: standard deviation]

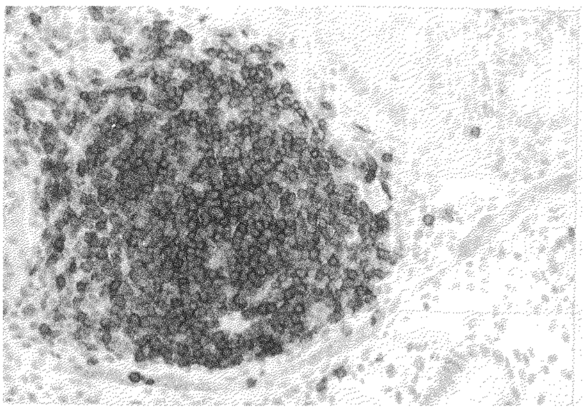

FIGURE 12 Lymphocyte aggregation consisting almost exclusively of B-lymphocytes ( x 700).

itive cells were found occasionally in intraepithelial and subepithelial layers of endometrium which were distinguished from the epithelum. Quantitative features of HLA-DR positive cells were similar in both groups of patients.

Beginning with the 5th day of menstrual cycle the number of HLA-DR positive cells decreased in both investigated groups from the mean value of 470 HLA-DR positive cells per $mm^2$ to 200 cells per $mm^2$ on day 12 of the cycle. After the 25th day of the menstrual cycle we noted a slight increase of the number of positive cells to the mean value of 300 cells per $mm^2$. There were no significant difference pattern discernable.

### *HLA-DR Expression in Superficial Epithelium*

No differences were found in distribution and intensity of HLA-DR expression of superficial epithelia in both the control and endometriosis groups. HLA-DR positive superficial cells were detected in the epithelial samples of both groups during the entire menstrual cycle, however, their amount decreased continuously after preovulatory maximum towards the end of the cycle (Fig. 13B).

### *HLA-DR Expression in Glandular Epithelium (Figure 13C)*

No significant cyclic dependence was found in the HLA-DR positivity of glandular epithelium in both the endometriosis and control groups. In the follicular phase of the cycle HLA-DR positivity of glandular epithelium was observed in more than half of the slides. The majority of epithelial cells demonstrated the equal low colour inten-

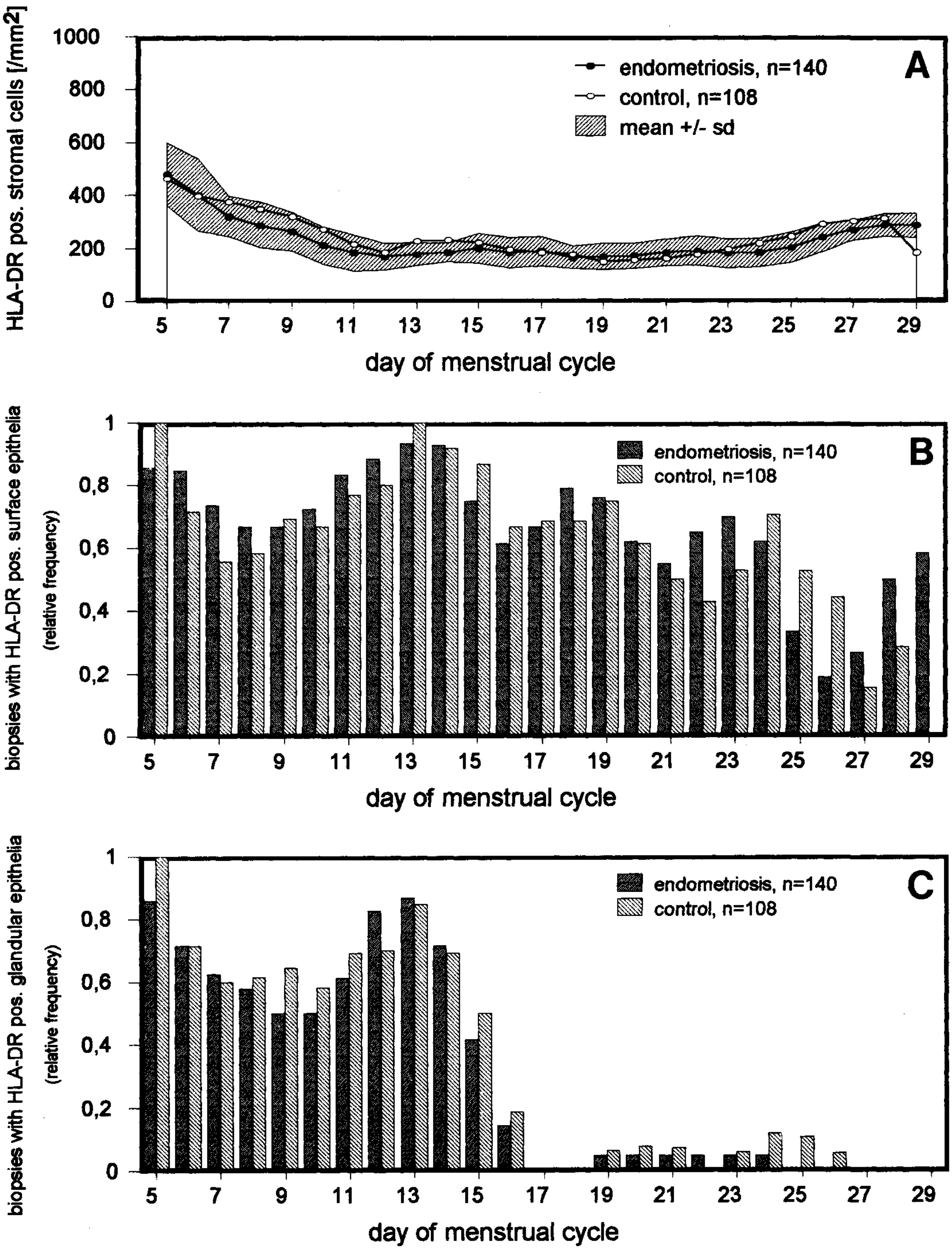

FIGURE 13 HLA-DR positive stromal cells (A) in the endometrium of women with endometriosis. Occurrence of HLA-DR positive surface (B) and glandular (C) epithelia in endometrial biopsies of women with endometriosis. [sd: standard deviation]

sity. After the 13th day of menstrual cycle the number of the slide with HLA-DR positivity in glandular epithelium dramatically decreased. The antibody positive epithelial cells were intensively coloured and restricted mainly to the lumen. Figure 14 shows the characteristic features of HLA-DR positive cells in the endometrium.

### Macrophages (Figure 15)

The occurrence and distribution of macrophages in endometrium during the menstrual cycle were approximately similar in both investigated groups. The changes in cell-quantity studied with the help of macrophages antibodies Ki-Mlp, Ki-M6 and Ki-M8 were negligable ($P > 0.05$), allowing to be described together. At the beginning of the menstrual cycle the average number of the observed macrophages varied between 150–200 cells per $mm^2$ and slowly decreased to 100 +/− 10 cells per $mm^2$ on day 10–11 of the menstrual cycle.

In glandular epithelium they were detected mainly as subepithelial cells. In lymphocyte aggregation macrophages were found mostly at margins, they occured usually as scattered single cells in lymphocyte aggregations (Fig. 16).

### Lymphocyte Aggregations

Lymphocytes aggregations were observed during the entire menstrual cycle in both groups of

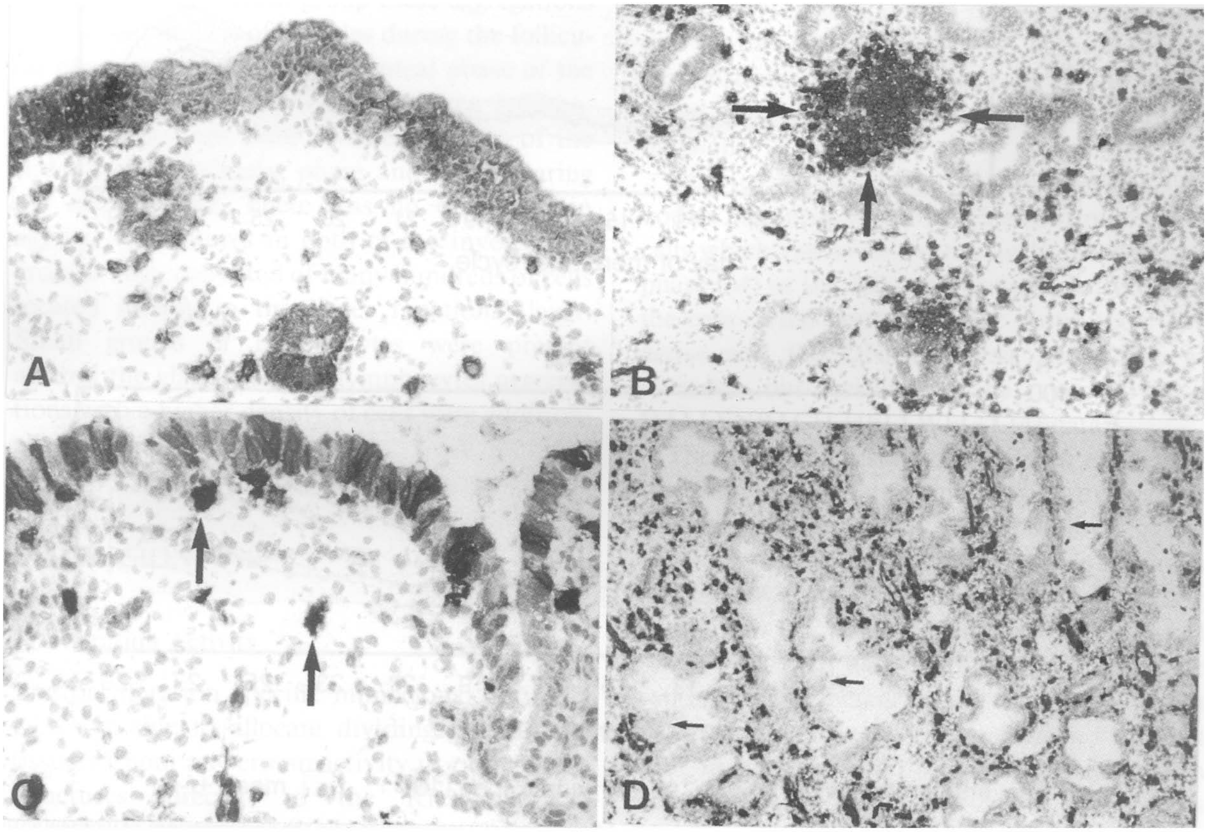

FIGURE 14 Homogenous HLA-DR expression in glandular and surface epithelia in early proliferative phase (A, × 700). Lymphocyte aggregations (arrows) showing intensive HLA-DR expression (B, × 350, cycle day 16). On cycle day 23 (C, × 700) single surface epithelia and scattered cells in the stroma (arrows) labelling for HLA-DR. In late secretory phase (DE, × 175) stromal HLA-DR expression contrasting to negative glands (arrows).

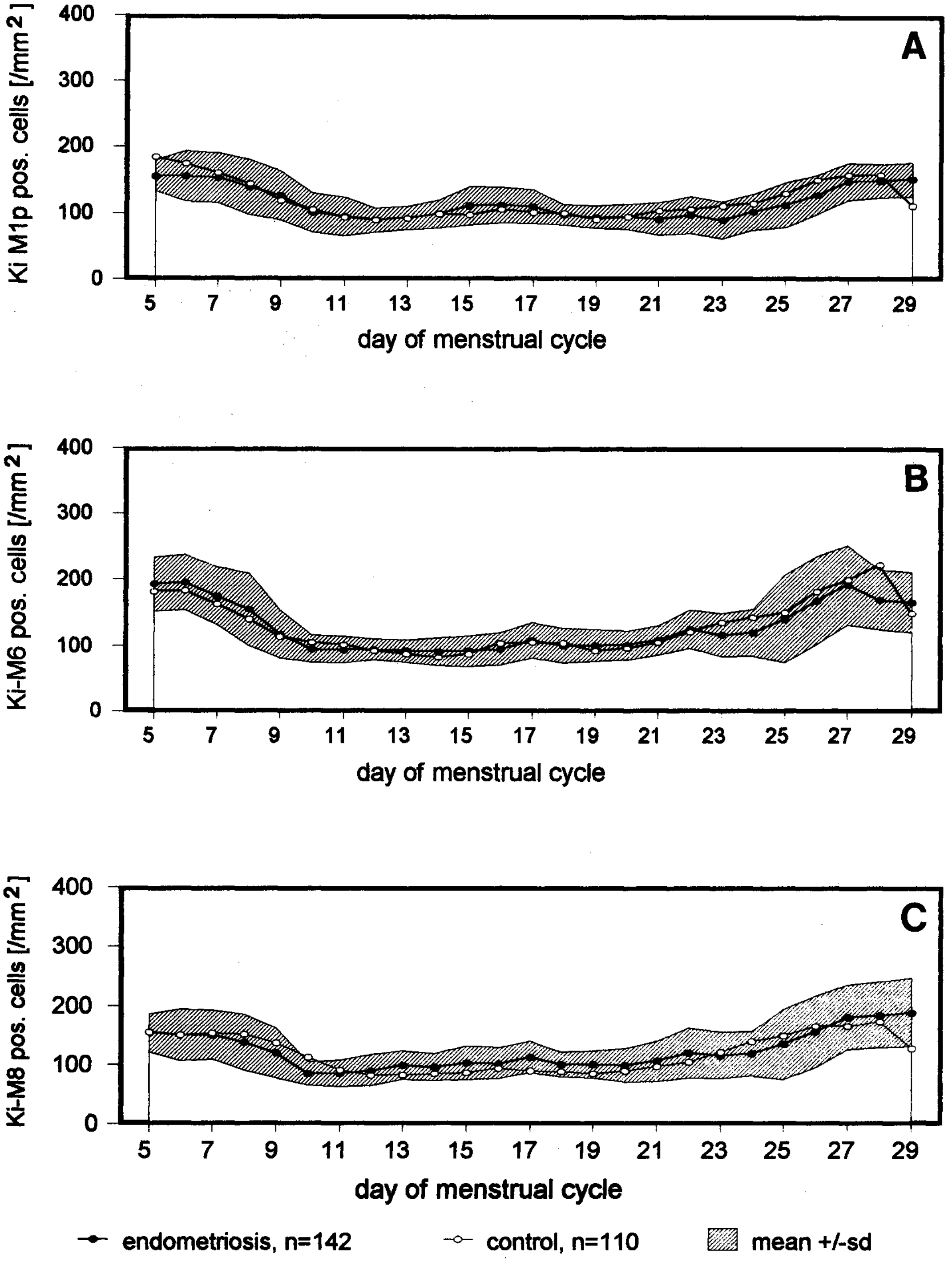

FIGURE 15 Ki-Mlp positive (A), Ki-M6 positive (B) and Ki-M8 positive (C) macrophages in the endometrium of women with endometriosis througout the menstrual cycle. [sd: standard deviation]

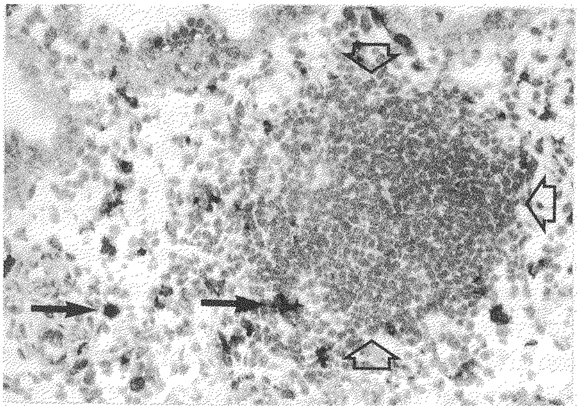

FIGURE 16 Macrophages scattered in endometrial stroma (arrows) and at the margin of a lymphocyte follicle (open arrows) in secretory phase ( × 700).

patients. In the control group these aggregations were found in 22% of samples during the follicular phase and in 10% during luteal phase of the cycle. In the endometriosis group the lymphocytes aggregations were revealed in 27% of the slides during follicular phase and in 6% during the luteal phase. There was no difference in aggregation features in both of the investigated groups. They consisted of many hundreds of cells located mainly in the lower functional layer. Small groups of lymphocytes were present around the glands. Larger lymphocytes aggregations with a higher density of cells were additionally detectable.

### DISCUSSION

### Proliferation Activity

The proliferation specific monoclonal antibody Ki-S3 is able to allocate dividing cells in the tissue without any crossreactivity to cytoplasmic structures directed to the Ki-S7 antigen (GERDES *et al.* 1984). The results with the proliferation specific monoclonal antibody Ki-S3 clearly monitor the cycle derived changes in the proliferation activity of the cellular components of the endometrium. In addition it becomes evident that considerable differences exist in the proliferation behavior of various cell types during the menstrual cycle. As expected the superficial and glandular epithelium proved to show high proliferation activity starting from the 5th day of menstrual cycle and keeps this level of activity up to ovulation without a recognizable maximum. After the 23th–25th days of the menstrual cycle the superficial and glandular epithelium demonstrated no more proliferative activity.

According to DALLENBACH-HELLWIG [4] the mitosis and DNS level, as demonstrated by light microscopy, reaches the maximum in epithelium and stroma at the time of ovulation. Then the dividing activity decreases and after day 18–19 of the cycle in glandular epithelium and to the 20th–21th days in stromal cells it demonstrates no mitotic activity at all. In the luteal phase there is an increase of mitotic division.

Due to the fact that Ki-S3 monoclonal antibodies not only allow to allocate the proliferating cells which appear in the relatively short mitotic phase (M) of the cell cycle but also to recognize the cells of the premitotic (G2 postmitotic (G1) phase and the DNA-synthesis phase (S), we consider our results to be supportive of DALLENBACH-HELLWEG's [4] data.

The described results of the study concerning distribution and pattern of proliferative cell activity in patients with endometriosis and in control-patients include data of immunohistochemical studies of endometrium in healthy women [16,23]. The immunohistochemical studies of occurence and distribution of steroid receptors in the female genital tract [19] demonstrate the coherence of estrogen receptors (ER) as well as growth and differentiation of endometrium. Using semiquantitative immunohistochemical methods PRESS *et al.* [19] report that distribution and intensivity of certain concentrations of ER in endometrium reflects the proliferation features of this tissue.

The maximal ER content in all layers of endometrium was found at the moment of ovulation which corresponded to a high proliferative activity of stromal cells reflecting the activity of functional and basal layers. At the secretory phase the ER content declined. These findings correlate with mitotic rest of glandular epithelium but not with the status of numerous available actively dividing cells in the stroma. The described difference can be attributed to the cycle dependent receptors and to epidermal growth factors (EGF) whichappear to be active mitogens.

Some authors report of a marked elevated quantity of EGF-receptors at the follicular phase of the cycle with its maximum in the preovulatory moment. Other investigators [18] describe in their immunohistochemical study of EGF-receptors in the endometrium of healthy women and in patients with endometriosis the uniform expression of these receptors without any cycle dependent alterations.

The immunohistochemical study of leucocytes (CD45, CD11c, Cb3) with monoclonal antibody Ki-67 demonstrated the existence of actively dividing lymphocytes in lymphocytes aggregations and as individual cells in the endometrial stroma [16,21]. The number of these cells increased dramatically during the luteal phase of the cycle. Thus, the proliferative activity of endometrial stromal cells at the end of menstrual cycle co-exists with an increase of dividing activity of immunocompetent cells and is partially attributed to the occurence of these cells.

### T-Lymphocytes

Different T-lymphocytes subgroups existing at variable numbers during the entire menstrual cycle were revealed as single cells as well as intraepithelial or subepithelial cells in aggregations. The dynamic patterns of cell numbers were nearly identical during the menstrual cycle in both the control and endometriosis groups. The highest cell quantity was demonstrated by the pan-lymphocytes-marker anti-Leu4 (CD3). The features the subgroups demonstrated were two maximas — in the early proliferation phase and at the end of the secretory phase with almost a horizontal curve in between. The most striking changes of cell numbers were found at the early and mid proliferation phases. The quantity of different T-lymphocytes expressed the slight fluctuations during the second half of the cycle which did not exceed +/− 1- cells per $mm^2$ The relatively high cell number at the beginning of menstrual cycle may be explained anatomically. The functional layer of endometrium has a thickness from 0.5 to 4 mm. Thus at the beginning of the proliferation phase a biopsy of the deepest part of endometrium with a high cell density may be taken. It also may be valid for a more frequent occurence of lymphocyte aggregations in the proliferative phase as compared to the secretory phase. CD4 positive and CD8 positive cells during the early proliferation phase revealed almost an equal quantity, and the ratio of CD4 to CD8 was almost 1:1. After the common decrease of cells numbers in the proliferative phase the number of CD4 positive cells continuously decreased while the level of CD8 positive cells was relatively constant. We could observe a predominance of CD8 positive T-lymphocytes with the ratio of CD4 : CD8 between 1:2 and 1:4. This predominance of CD8 positive suppressor lymphocytes in comparison with CD4 positive T-helpers was found in both groups of the patients. These data correspond to the ones of VIGANO *et al.* [24] concerning women with endometriosis. The study of T-lymphocytes in the thoracic duct of men and women [3] demonstrated that only CD8 positive lymphocytes, but not CD4 positive ones possessed estrogen receptors. Evidently, there is the preference of these cells in estrogen sensitive tissues. MORRIS *et al.* [15] declared the ratio of intraepithelial CD4 positive T-lymphocytes and CD8 positive T-lymphocytes occurence as high as 1:4. These data correspond to the results of our old study [2] which suggested almost the same ratio for women with endometriosis. It is difficult to conclude if the noted increased ratio was due to the presence of endometriosis or only infertility. We may explain it by the fact that most T-lymphocytes in the endometrium act as TCR-1-lymphocytes which make up only 5% of all T-lymphocytes but are found mainly in skin and mucosa. They are CD3 positive and can express CD8-epitopes but not CD4 epitopes [3,12].

The quantity of Leu-7 positive cells in the patients with endometriosis and in the controls were practically undistinguishable. LYDYARD and GROSSI in their study of immunological properties of natural killer cells (NK cells) proved the presence of small numbers of Leu-7 positive NK cells in endometrium which were the subject to discrete cyclic fluctuations. The analogous results concerning the endometrium of healthy women were described by KAMAT and ISAACSON 1987, KING *et al.* 1988 [9,10].

Immunohistochemical double colouring proves that proliferation activity inendometrial stroma at the end of menstrual cycle is not only attributed to stromal cells but also to leucocytes [23]. Proliferating activity of granulocytes also exists in proliferating T-lymphocytes. Immunohistochemical double colouring for proliferation specific antibodies and other NK-cell-markers may give us additional information whether the endometrium of endometriosis patients and infertile patients gives different results in respect to proliferation activity of granulocytes.

**B-Lymphocytes**

Only few single B-lymphocytes were found in the endometrium of investigated patients. Their number increased considerably in some lymphocyte aggregations forming areas within these aggregations almost exclusively consisting of B-lymphocytes. Our findings coincide with immunochemical investigations of endometrium in healthy women [15,9,11].

**Anti HL-DR**

The investigation of HLA-DR revealed no difference in expression of this antigen during the menstrual cycle in different layers of endometrium in patients of both groups.

LAGUENS *et al.* [11] described in their study the cyclic dependent increase of HLA-DR positive cells in endometrial stroma from 13–25% in the proliferation phase to 16–43% at the late secretion phase. The macrophages HLA-DR positivity input was estimated as 1/10 to 1/2 of HLA-DR positive cells. No HLA-DR positive cells and macrophages were found in the endometrium of post-menopausal women. Therefore HLA-DR positive cells may be the subject to direct or indirect hormonal influence.

The results of other investigations demonstrated no increase of HLA-DR positive stromal cells during the menstrual cycle [22]. A possible explanation of the discrepancy of results in the present study and the others mentioned may be the small number of investigations and matched ages of patients in those investigations.

In tissue culture slide [22] it was noted that the addition of either T-lymphocytes or — interferon to human endometrium culture may have a dose dependent influence on HLA-DR expression and proliferation activity of endometrial cells. The authors consider this T-cells mediated effect as possible explanation for the reinforced proliferation of functional endometrium. One of the important points of these observations is the possibility of local influence on the hormone dependent tissue by cells of the immune system. It is also possible to assume the interaction of the immune system due to the existence of HLA-DR antigens in the endometrial stromal and epithelial cells.

**Macrophages**

Macrophages in endometrium were mainly homogenously distributed in the endometrial stroma. Occasionally they were found in intraand subepithelium. The importance of macrophages in the endometrium in connection with endometriosis and associated infertility remains unclear. At the same time some investigators report of an increased level of macrophages and macrophage activity in the peritoneal fluid of patients with endometriosis [7,8,13]. Certainly we [13] described this fact in earlier studies and are still investigating on MCSF the macrophage stimulation factor activity in ectopic endometrium.

We failed to find any study in the literature concerning the occurence of macrophages in the endometrium of women with endometriosis. As far as women without endometriosis are concerned some authors report on the occurence of macrophages during the menstrual cycle [10]. Others found either an elevated number of macrophages at the secretory phase [11] or an increased dividing activity during the secretory phase [21,24].

### Follicle Like Lymphocyte Aggregations

The aggregations were mainly located in the lower functional and basal layers of endometrium consisting of B-lmyphocytes in the central area, and of T- lymphocytes which were mostly represented in the margin areas. Almost all aggregations of lymphocytes expressed HLA-DR positivity. The dividing activity was marked during the entire menstrual cycle. Similar data concerning the occurence, distribution and composition of lymphocytes aggregations in the endometrium of women with endometriosis are reported which are based on the results of small groups of patients without matched age [21,15,9].

In conclusion, our study based on the comparison of some functions of endometrial cells of infertile women with endometriosis and without endometriosis demonstrates the high level of proliferative activity of stromal cells at the end of menstrual cycle. These data is confirmed by the vitality and potential ability of endometrial cells to survive in the peritoneal cavity.

Answering the question if the proliferative activity of endometrial cells varies in women with and without endometriosis we can clearly state that there is no difference in endometriosis and non endometriosis patients.

The lack of any difference in proliferative activity in the control and endometriosis groups might be an evidence that the cause for deriving endometriosis from endometrial cells probably should be attributed to the responsibility of the extrauterine milieu.

The observed quantitative differences in occurence, distribution, number and correlation among different subpopulations of T- and B-lymphocytes macrophages, lymphocytes aggregations and the expression of HLA-DR positivity demonstrated in both groups quite clearly a cyclic dependence. It provides us with evidence of a tight connection between ovarian function and local functions of immunocompetent cells and confirms the characteristics of endometrium as mucosa associated lymphatic tissue.

### *References*

[1] American Fertility Society. Revised American Fertility Society Classification on Endometriosis. *Fertil. Steril.* 1985; **43**: 351–352.

[2] Bonatz, G., Hansmann, M., Buchholz, F., Mettler, I., Radzun, H., Semm, H.: Macrophage- and lymphocyte-subtypes during different phases of the ovarian cycle. *Int. J. Gynecol. Obstet.* 1992; **37**: 29–36.

[3] Cohen, J., Danel, L., Cordier, G., Salz, S., Revillard, L.: Sex steroid receptors in peripheral T-cells: absence of androgen receptors and restriction of estrogen receptors to OKT8-positive cells. *J. Immunol.* 1983; **131**: 2767–2771.

[4] Dallenbach-Hellweg G.: Histopathologie des Endometriums. 3. Auflage, Springer Verlag, Berlin, Heidelberg, New York, Tokyo, 1981.

[5] Dmowski, W., Gebel, M., Rawlins, R.: Immunologic aspects of endometriosis. *Obstet. Gynecol. Clin. North Am.* 1989; **16**: 93–103.

[6] Gerdes, J., Lemke, H., Baisch, H., Wacher, H., Schwab, U., Stein, H.: Cellcycle analysis of a cell proliferation-associated human nuclear antigen defined to the monoclonal antibody Ki-S7. *J. Immunol.* 1984; **133**: 1710–1715.

[7] Halme, J., Becker, S., Haskill, S.: Altered maturation and function of peritoneal macrophages: possible role in pathogenesis of endometriosis. *Am. J. Obstet. Gynecol.* 1987; **156**: 783–789.

[8] Haney, A., Misukonis, M., Weinberg, J.: Macrophages and infertility: oviductal macrophages as potential mediatgors of infertility. *Fertil. Steril.* 1983; **39**: 310–315.

[9] Kamat, B., Isaacson, P.: The Immunocytochemical distribution of leucocytic subpopulations in human endometrium. *Am. J. Pathol.* 1987; **127**: 66–73.

[10] King, A., Wellings, V., Gardner, L., Loke, Y.: Immunocytochemical characterization of the unusual large granular lymphocytes in human endometrium throughout the menstrual cycle. *Hum. Immunol.* 1988; **24**: 195–205.

[11] Laguens, G., Goni, J., Laguens, M., Goni, J. M., Laguens, R.: Demonstration and characterization of HLA-DR positive cells in the stroma of human endometrium. *J. Reprod. Immunol.* 1990; **18**: 179–186.

[12] Lydyard P., Grossi, C.: The lymphoid system. In: Roitt, I., Brostoff, J., Male, D. (Hrsg). *Immunology*, 2. Teil, p. 31–39. Churchill Livingstone, Edinburgh, London, Melbourne.

[13] Mettler, L.: Immunophanotyp von Endometrium und Endometriose. *Endometriose* 1989; **3**: 64–69.

[14] Mettler, L., Giesel, H. and Semm, K.: Treatment of Female Infertility Due to Tubal Obstruction by Operative Laparoscopy. *Fertil. and Steril.* 1979; Vol 32, No. 4.

[15] Morris, H., Edwards, J., Tiltman, A., Emms, M.: Endometrial lymphoid tissue: an immunological study. *J. Clin. Pathol.* 1985; **38**: 644–652.

[16] Pace, D., Morrison, L., Bulmer, J.: Proliferative activity of the endometrial stromal granulocytes throughout the menstrual cycle in and early pregnancy. *J. Clin. Pathol.* 1989; **42**: 35–39.

[17] Parwaresch, M. R.: Personal communication, 1995.

[18] Prentice, A., Thomas, E., Wedell, A., McGill, A., Randall, B., Horne, C.: Epidermal growth factor receptors expression in normal endometrium and endometriosis: an immunohistochemical study. *Br. J. Obstet. Gynecol.* 1992; **99**: 395–398.

[19] Press, M., Nousek-Goeble, N., Bur, M., Greene, G.: Estrogen receptors localization on female genital tract. *Amer. J. Pathol.* 1989; **123**: 280–292.

[20] Steel, R., Dmowski, W., Marmer, D.: Immunologic aspects of human endometriosis. *Am. J. Reprod. Immunol.* 1984; **6**: 33–36.

[21] Tabidzadeh, S.: Proliferative activity of lymphoid cells in human endometrium throughout the menstrual cycle. *J. Clin. Endocrin. Metab.* 1990; **70**: 437–443.

[22] Tabibzadeh, S.: Induction of HLA-DR expression in endometrial epithelial cells by endometrial T-cells: potential regulatory role of endometrial T-cells in vivo. *J. Clin. Metabol.* 1991; **73**: 1352–1358.

[23] Tabibzadeh, S., Poubouridis, D.: Expression of leucocyte adhesion molecules in human endometrium. *Am. J. Clin. Pathol.* 1990; **93**: 183–189.

[24] Tabidzadeh, S., Satyaswaroop, P., Rao, P.: Antiproliferative effect of interferon — in human endometrial cells in vitro: potential local growth modulatory role in endometrium. *J. Clin. Endocrinol. Metab.* 1988; **67**: 131–138.

[25] Troche, V., O'Connor, D., Schandies, R.: Measurement of human epidermal growth factor receptor in the endometrium during menstrual cycle. *Am. J. Obstet. Gynecol.* 1991; **165**: 1499–1503.

[26] Vigano, P., Vercellini, P., DiBlasio, A.: Deficient anti-endometrium pymphocyte-mediated cytotoxicity in patients with endometriosis. *Fertil. Steril.* 1991; **56**: 894–899.

[27] Weinberg, J., Haney, A., Xu, F., Ramakrishnan: Peritoneal fluid and plasma levels of human macrophages colony stimulation factor in relation to peritoneal fluid content. *Blood* 1991; **2**: 513–516.

